# Brimonidine is Neuroprotective in Animal Paradigm of Retinal Ganglion Cell Damage

**DOI:** 10.3389/fphar.2021.705405

**Published:** 2021-07-21

**Authors:** Federica Conti, Giovanni Luca Romano, Chiara Maria Eandi, Mario Damiano Toro, Robert Rejdak, Giulia Di Benedetto, Francesca Lazzara, Renato Bernardini, Filippo Drago, Giuseppina Cantarella, Claudio Bucolo

**Affiliations:** ^1^ Department of Biomedical and Biotechnological Sciences, Section of Pharmacology, University of Catania, Catania, Italy; ^2^ Department of Ophthalmology, Jules Gonin Eye Hospital, Fondation Asile des Aveugles, University of Lausanne, Lausanne, Switzerland; ^3^ Department of Ophthalmology, University of Zurich, Zurich, Switzerland; ^4^ Chair and Department of General and Pediatric Ophthalmology, Medical University of Lublin, Lublin, Poland

**Keywords:** neuroprotection, retinal ganglion cells, ischemia-reperfusion, brimonidine, PERG

## Abstract

To investigate the neuroprotective effect of brimonidine after retinal ischemia damage on mouse eye. Glaucoma is an optic neuropathy characterized by retinal ganglion cells (RGCs) death, irreversible peripheral and central visual field loss, and high intraocular pressure. Ischemia reperfusion (I/R) injury model was used in C57BL/6J mice to mimic conditions of glaucomatous neurodegeneration. Mouse eyes were treated topically with brimonidine and pattern electroretinogram were used to assess the retinal ganglion cells (RGCs) function. A wide range of inflammatory markers, as well as anti-inflammatory and neurotrophic molecules, were investigated to figure out the potential protective effects of brimonidine in mouse retina. In particular, brain-derived neurotrophic factor (BDNF), IL-6, tumor necrosis factor-related apoptosis-inducing ligand (TRAIL) and its death receptor DR-5, TNF-α, GFAP, Iba-1, NOS, IL-1β and IL-10 were assessed in mouse retina that underwent to I/R insult with or without brimonidine treatment. Brimonidine provided remarkable RGCs protection in our paradigm. PERG amplitude values were significantly (*p* < 0.05) higher in brimonidine-treated eyes in comparison to I/R retinas. Retinal BDNF mRNA levels in the I/R group dropped significantly (*p* < 0.05) compared to the control group (normal mice); brimonidine treatment counteracted the downregulation of retinal BDNF mRNA in I/R eyes. Retinal inflammatory markers increased significantly (*p* < 0.05) in the I/R group and brimonidine treatment was able to revert that. The anti-inflammatory IL-10 decreased significantly (*p* < 0.05) after retinal I/R insult and increased significantly (*p* < 0.05) in the group treated with brimonidine. In conclusion, brimonidine was effective in preventing loss of function of RGCs and in regulating inflammatory biomarkers elicited by retinal I/R injury.

## Introduction

Glaucoma is an optic neuropathy characterized by retinal ganglion cells (RGCs) death, irreversible peripheral and central visual field loss and high IOP (Bucolo and Drago, 2011). Currently, six main classes of topical drugs are available; they include beta-blockers, carbonic anhydrase inhibitors, prostaglandin derivatives, sympathomimetics, miotics, and Rho-kinase inhibitors. For neovascular glaucoma the therapeutic approach could be different, on this regards it is worth of note that anti-VEGF agents, used in clinical practice, such as ranibizumab, bevacizumab and aflibercept are considerably different in terms of molecular interactions when they bind with VEGF (Platania et al., 2015). Brimonidine is an α2_A_-adrenergic receptor agonist, approved for lowering intraocular pressure (IOP) in patients with open-angle glaucoma. Although α2_A_ receptors have been identified in the RGCs, the mechanisms by which α2_A_ agonists exert neuroprotection are not well-established. There are many controversial studies on brimonidine and its effects to preserve retinal tissue. Some non-clinical findings have demonstrated that brimonidine possess retinal protective action (Lambert et al., 2011; Nizari et al., 2016; Marangoz et al., 2018). However, to date, clinical trials have failed to translate into similar efficacy in humans. Recently, a Cochrane systematic review (Sena and Lindsley, 2017) showed that although one clinical trial found less visual field loss in the brimonidine-treated group, the evidence was of such low certainty that it is not possible draw conclusions from this only finding. Incidentally, the authors concluded that further clinical research is needed to determine whether brimonidine may be beneficial for individuals with glaucoma. More recently, a systematic review and meta-analysis concluded that the clinical evidence of neuroprotective effect of brimonidine is inconclusive and needs stronger support maybe with large double-blind randomized clinical trials (Scuteri et al., 2020). To shed light on these controversial studies we aimed to investigate topical brimonidine on a well-known *in vivo* paradigm of retinal damage. The neurodegenerative process in several eye diseases is characterized by progressive death of RGCs, optic nerve degeneration, and sometime blindness (Chou et al., 2020). RGCs degeneration is often associated to ischemia in central retinal artery occlusion and ischemic optic neuropathies (Kunimi et al., 2019). Remarkable insights in therapy for retinal ischemia have arisen through the investigation of rodent models of ischemia-reperfusion. Retinal ischemia–reperfusion (I/R) is an experimental model that triggers an inflammatory process eliciting a large number of detrimental molecules such as TNF, tumor necrosis factor-related apoptosis-inducing ligand (TRAIL) and ILs (Osborne et al., 2004; Wei et al., 2011; Dibas et al., 2018).

Gliosis, another critical event contributing to glaucoma pathogenesis, is a hallmark of retinal degeneration. Retinal reactive glia cells increased glial fibrillary acidic protein (GFAP)-immunoreactivity and ionized calcium binding adaptor molecule 1 (Iba1). It is well known that injury-induced gliosis in the optic nerve head and retina promote the death of RGCs due to over-release of pro-inflammatory mediators (Ganesh and Chintala, 2011). TRAIL mediates different neuroinflammatory responses (Huang et al., 2011). TRAIL and its receptors were found up-regulated in brain ischemia-reperfusion (Cui et al., 2010). The unmet medical need in glaucoma is mainly related to disease progression (RGC death) despite IOP control. In fact, glaucoma progression could be related to neurotrophins deprivation; interestingly, low serum levels of BDNF and nerve growth factor (NGF) were associated to early moderate stages of glaucoma. It is worth of note that the potential therapeutic value of neurotrophins to manage glaucoma is important, however the main point that damper the development of these factors as eye drops is related to the drug delivery issues (Bucolo et al., 2018). On this regards it could be useful develop a biodegradable deliver system in order to sustain prolonged pharmacological levels of drug into the back of the eye (Conti et al., 1997) even though topical formulation is ideal. Aim of the present study was to investigate the neuroprotective effects of brimonidine eye drops in a mouse model of retinal I/R damage. Pattern electroretinogram (PERG) analysis, the most specific non-invasive technique for electrophysiological assessment of RGCs activity, was used to evaluate the *in vivo* protection of RCGs function. Further, the retinal inflammatory profile after I/R insult with or without brimonidine treatment was investigated.

## Materials and Methods

### Animals

Male C57BL6/J mice (Charles River Laboratories, Italy) were housed in a temperature-controlled environment with free access to food and water during a 12-h light–dark cycle. All animals were treated according to the Principles for the Care and Use of Animals in Ophthalmic and Vision Research approved by the Association for Research in Vision and Ophthalmology. University of Catania (Italy) Ethics Committee approval #343.

### Ischemic-Reperfusion Retina Damage

Retinal ischemia/reperfusion has been used as a model of retinal injury and has been described in many rodent species (Osborne et al., 2004; Gustavsson et al., 2008; Ulbrich et al., 2017; Stankowska et al., 2019). A validated modified I/R model (Hartsock et al., 2016) (Hartsock et al., 2016) was used in the present study. Mice were anesthetized by intraperitoneal injection with tiletamine + zolazepam (60 mg/kg) and medetomidine (40 μg/kg) plus a topical instillation of 0.4% oxybuprocaine (Novesina®, Laboratoires Thea, Clermont-Ferrand, France). The animals were placed on a heating pad to prevent hypothermia during the experiment. A 32-gauge needle, connected with a reservoir containing PBS, was introduced into the anterior chamber through the cornea to increase intraocular pressure (up to 90 mm Hg). Retinal ischemia was confirmed by an observation of blanching of the anterior segment and arteries in the eye. Following 60 min of ischemia, the needle was removed to allow rapid reperfusion. Ocular formulation of brimonidine tartrate (2 mg/ml) was instilled (10 µL) 60 min before I/R and after reperfusion, twice in 2 h. The effect of brimonidine was evaluated after 72 h from I/R insult. Mice were euthanized after 72 h from I/R insult, the eyes were enucleated, and the retinas collected.

### Pattern Electroretinogram

**FIGURE 1:**
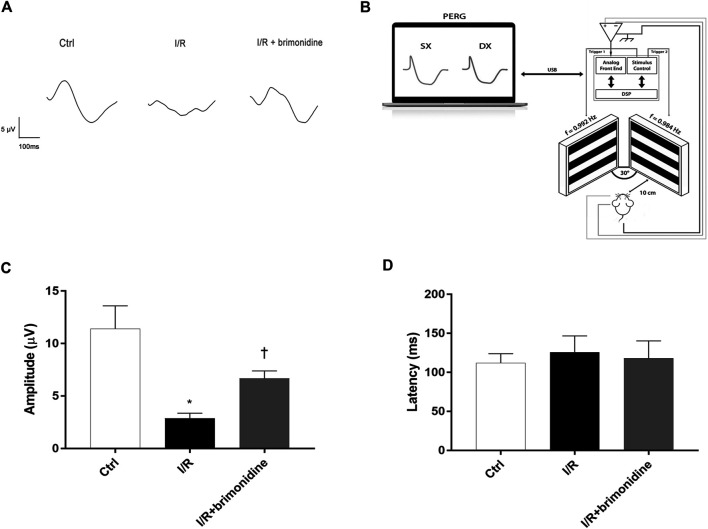
RGCs function assessment. **(A)** Representative PERG waveforms in C57BL6/J mice control, I/R and I/R plus brimonidine. **(B)** Mouse PERG recording layout. **(C)** Comparison between PERG amplitude values (µV) and latency values **(D)** of control, I/R and brimonidine treated mice. Brimonidine significantly counteracted RCGs loss of function induced by I/R injury, after 72 h, in mice retina. In each panel, bars represent the mean values and corresponding standard errors (±SD). One-way ANOVA analysis was performed followed by the Tukey post-hoc test. *p < 0.05 *vs.* Ctrl; ^†^ *p* < 0.05 *vs.* I/R.

As a sensitive measure of RGCs function we used the PERG (Chou et al., 2018). Anesthetized mice were transferred on a heating plate with the mouse superior incisor teeth hooked to a bite bar and the head gently restrained by two ear knobs. Body was kept at a constant temperature of 37°C using a feedback-controlled heating pad (TCAT-2LV, Physitemp Instruments, Inc., Clifton, NJ, United States). Two microliters of balanced salt solution (BSS) were topically applied to prevent corneal dryness. Simultaneous recordings of PERG response from both eyes were obtained using a common subcutaneous needle in the snout with a commercially available instrument (Jorvec Corp., Miami, FL, United States). Figure 1, panel B, shows the mouse PERG recording layout. Visual stimuli consisted of black-white horizontal bars generated on LED tablets and presented independently to each eye at 10 cm distance (56° vertical × 63° horizontal field; spatial frequency, 0.05 cycles/deg; 98% contrast; 800 cd/sqm mean luminance; left-eye reversal rate, 0.992 Hz; right-eye reversal rate, 0.984 Hz). Electrical signals recorded from the common snout electrode were averaged (>1,110 epochs), and PERG responses from each eye isolated by averaging at stimulus-specific synchrony. As previously described [17], PERG waveforms consisted of a positive wave (defined as P1) followed by a slower negative wave with a broad trough (defined as N2). Therefore, each waveform has been analyzed by measuring the peak-to-trough (P1-N2) amplitude defined as PERG amplitude and the time-to-peak of the P1 wave as PERG latency (Porciatti, 2015).

### Ribonucleic Acid (RNA) Extraction and Complementary Deoxyribonucleic Acid (cDNA) Synthesis 

Mice were sacrificed after 72 h from I/R and brimonidine treatment by cervical dislocation, eyes were enucleated, and retinas were isolated. The extraction of total RNA from mice retina samples was performed by using TRIzol Reagent (Invitrogen, Life Technologies, Carlsbad, CA) according to the manufacturer’s protocol. The A260/A280 ratio of the optical density of RNA samples (measured with Nanodrop spectrophotometer ND-1000, Thermofisher) was 1.95–2.01. cDNA was synthesized from 500 ng of RNA with a reverse transcription kit (SuperScript^™^ II Reverse Transcriptase, Invitrogen, ThermoFisher Scientific, Carlsbad, CA, United States).

### Quantitative Real-Time Polymerase Chain Reaction (RT-PCR)

RT-PCR was performed with the Rotor-Gene Q (Qiagen). The amplification reaction mix included Master Mix Qiagen (Qiagen QuantiNova SYBR Green Real Time-PCR Kit) and cDNA. For each sample, were made forty-five amplification cycles, in triplicate. Melting curve analysis confirmed the specificity of the amplified products. Results were analysed with the 2^−ΔΔCt^ method and expressed as fold change vs. control. Quantitative PCR experiments followed the MIQE guidelines. BDNF and IL-6 genes were analyzed by using specific primers purchased from Eurofin Genomics (Milan, Italy) and Qiagen (Milan, Italy) respectively. Gene expression levels were normalized with levels of a constitutively expressed gene (18S, Eurofin Genomics). Primer sequences are listed in Table 1.

**TABLE 1:** Primers used for RT-PCR.

Gene	Primer murine sequence/Catalogue number
18 s	Forward: 5′-GTT​CCG​ACC​ATA​AAC​GAT​GCC-3′
	Reverse: 5′-TGG​TGG​TGC​CCT​TCC​GTC​AAT-3′
BDNF	Forward: 5′-GTT​CGA​GAG​GTC​TGA​CGA​CG-3′
	Reverse: 5′-AGT​CCG​CGT​CCT​TAT​GGT​TT-3′
IL-6	Cat. No. QT00098875

### Tissue Homogenization and Protein Extraction

Proteins were extracted from the retina samples with RIPA lysis buffer containing protease inhibitor cocktail, EDTA-free (Sigma, Inc.) by first sonicating for 20 s, and then centrifuging for 15 min at 14,000 rpm at 4°C. The supernatant was collected in new tubes and placed on ice. The protein concentration was measured using the Pierce^™^ Coomassie Protein Assay Kit (ThermoFisher, Monza, Italy).

### Western Blot

Equal amounts of protein (30 µg) were resolved by 8–12% SDS-PAGE gels and transferred onto Hybond ECL nitrocellulose membranes (GE Healthcare, Little Chalfont, United Kingdom). Membranes were blocked for 1 h at room temperature with 5% nonfat dry milk in phosphate-buffered saline plus 0.1% Tween 20 (PBS-T) and were then probed overnight with the following appropriate primary antibodies: rabbit anti-TRAIL (1:200, ab2435; Abcam, Cambridge, United Kingdom); rabbit anti-DR5 (1:500, ab8416; Abcam Cambridge, United Kingdom); mouse anti-GFAP (1:500, ab3670; Cell Signaling Technology, Inc., Danvers, MA, United States); rabbit anti-Iba1 (1:1000, PA5-27436; Thermo Fisher Scientific Italy, Rodano, Milan, Italy); rabbit anti-TNF-α antibody (1:1000, NB600-587; Novus Biologicals, Milan, Italy); rabbit anti-IL10 antibody (1:500, 250,713; Abbiotec, San Diego, CA, United States); rabbit NOS2 (1:250, sc-651; Santa Cruz Biotechnology Inc., Santa Cruz, CA, United States); mouse anti-IL-1β (1:250, sc-52012; Santa Cruz Biotechnology Inc., Santa Cruz, CA, United States). Then, the membranes were washed with PBS-T, and probed with the appropriate horseradish peroxidase-conjugated anti-rabbit or anti-mouse IgG antibody (GENA934, GNENA931; Amersham Life Science, Buckinghamshire, United Kingdom) for 1 h at RT. Beta-Tubulin (1:500, sc5274; Santa Cruz Biotechnology Inc., Santa Cruz, CA, United States) was used as control to validate the amount of protein loaded in the gels. After washing with PBS-T, protein bands were visualized by enhanced chemiluminescence (Thermo Fisher Scientific, Milan, Italy) and scanned with the iBright FL1500 Imaging System (Thermo Fisher Scientific, Milan, Italy). Densitometric analysis of band intensity was done on immunoblots by using IMAGE J software (https://imagej.nih.gov/ij/).

### Statistical Analysis

Statistical analysis was performed using GraphPad Prism Software, version 8 (GraphPad Software, Inc., San Diego, CA, United States). PERG amplitude and latency were analyzed for significance with one-way ANOVA followed by Tukey test for multiple comparisons. For single comparisons, Student’s t test was applied. *p* values ≤ 0.05 were considered statistically significant. Data are plotted as mean ± SD.

## Results

### Retinal Ganglion Cells Function was Ameliorated by Brimonidine TreatEment

Figure 1 shows that 72 h after I/R, RGCs function, measured with PERG, was reduced by more than 50%. This effect was significantly attenuated by brimonidine treatment. Representative PERG waveforms recorded from the eyes in each group are shown in Figure 1A. PERG amplitudes of control group, I/R group, and I/R plus brimonidine group were compared as shown in Figure 1C. The average value of control PERG amplitude was 13.8 μV in agreement with previous studies on wild type mice (Romano et al., 2020). No significant changes were observed in terms of latency in all groups (Figure 1D) as expected considering the short time after the injury, whereas the average PERG amplitude of I/R mice was significantly (*p* < 0.05) reduced compared to the control retina. Worth of note, the average value of PERG amplitude of I/R brimonidine–treated mice, was significantly (*p* < 0.05) higher when compared with I/R, suggesting a protection of RGC function.

### Neuroprotective and Anti-inflammatory Effect of Brimonidine in I/R-Injured Mice

**FIGURE 2:**
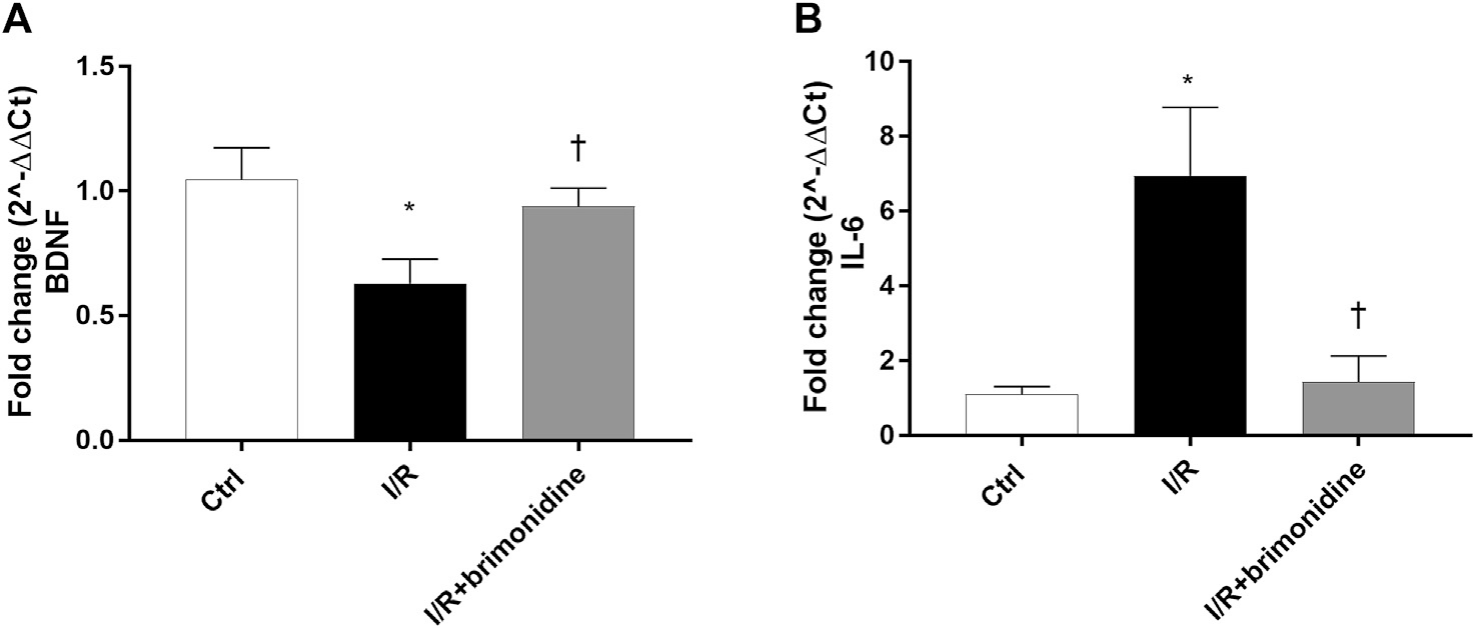
BDNF and IL-6 mRNA expression in mice retina. Brimonidine treatment maintained BDNF **(A)** mRNA levels close to control group, in comparison to I/R injured mice. Furthermore, brimonidine reverted the up-regulation of IL-6 **(B)** elicited by I/R injury. The mRNA levels were evaluated by RT-PCR; values represent the mRNA fold changes relative to 18 S used as housekeeping gene. Values are reported as a mean ± SD (*n* = 5). One-way ANOVA analysis was performed followed by the Tukey post-hoc test. *p < 0.05 *vs.* Ctrl; ^†^ *p* < 0.05 vs. I/R.

**FIGURE 3:**
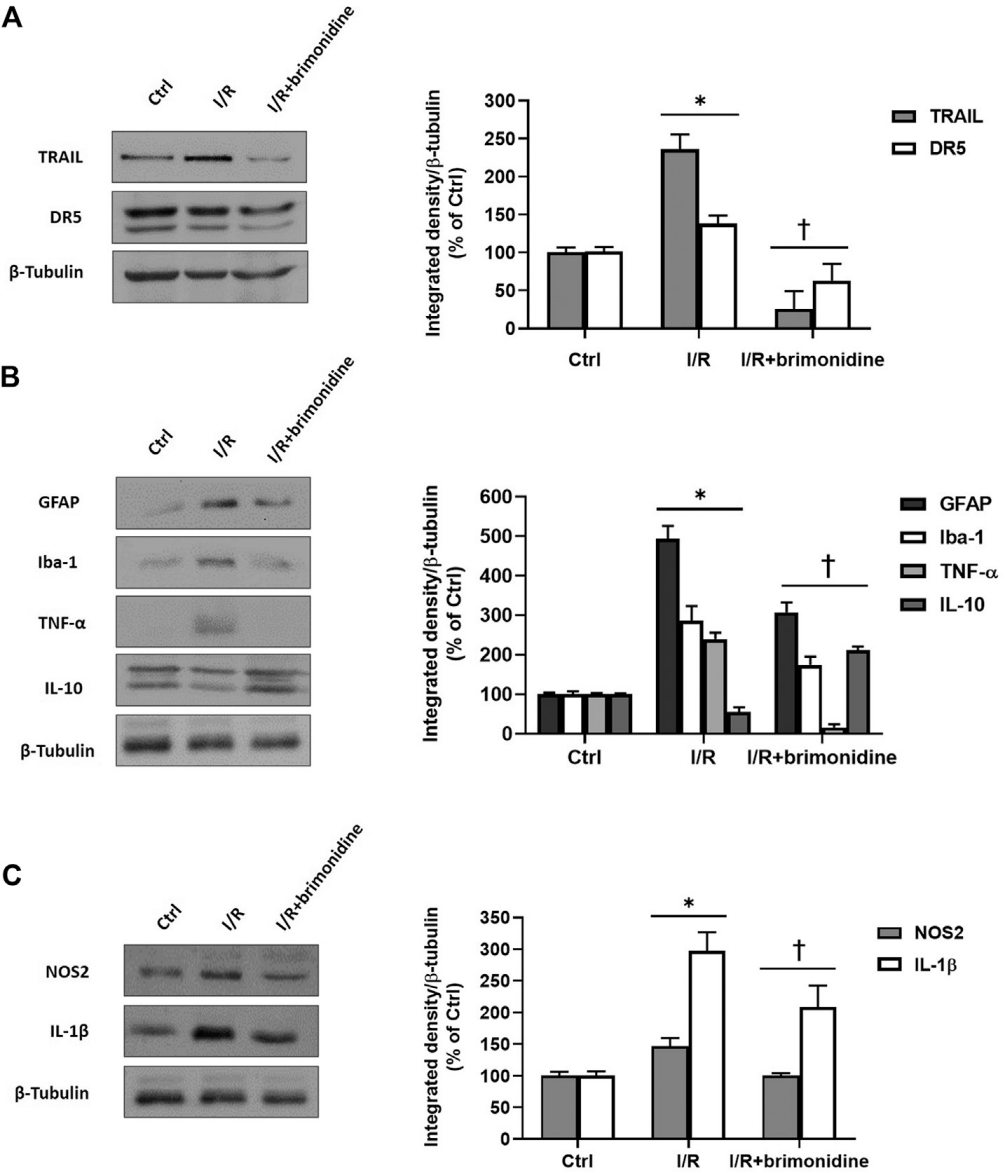
Western Blot. **(A)** TRAIL and DR5 protein levels in control, I/R and brimonidine-treated mice retina; **(B)** GFAP, Iba-1, TNF-α and IL-10 proteins in mice retina w or w/o brimonidine; **(C)** NOS2 and IL-1β proteins in mice retina w or w/o brimonidine. Values represent protein expression relative to β-tubulin, used as housekeeping protein. Values are reported as mean ± SD (*n* = 5). One-way ANOVA analysis was performed followed by the Tukey post-hoc test. *p < 0.05 *vs.* Ctrl; ^†^ *p* < 0.05 vs. I/R.

I/R injury significantly (*p* < 0.05) downregulated the mRNA expression of BDNF in mice retina, while treatment with brimonidine maintained BDNF mRNA levels close to the control group values, with a significant difference (*p* < 0.05) compared to I/R group (Figure 2A). Furthermore, I/R insult elicited significant (*p* < 0.05) increase of IL-6 mRNA levels, that was significantly (*p* < 0.05) reduced by brimonidine treatment (Figure 2B). To better investigate the anti-inflammatory effect of brimonidine treatment on mice retina, we analyzed different inflammatory mediators. In particular, we found that protein levels of TRAIL and its receptor DR-5, were significantly (*p* < 0.05) higher in I/R injured retina, while brimonidine treatment significantly (*p* < 0.05) reduced the expression of both proteins (Figure 3A). In consideration of the well-known involvement of retinal activated microglia, astrocytes and Muller glial cells in glaucoma, we assessed retinal Iba1 and GFAP expression, which were significantly (*p* < 0.05) increased after I/R injury (3-fold and 5-fold, respectively) compared to control (Figure 3B). The effect of brimonidine was demonstrated by the remarkable reduction of Iba1 and GFAP levels in the retinal tissue (Figure 3B). Furthermore, I/R insult significantly (*p* < 0.05) increased retinal levels of pro-inflammatory cytokines such as TNF-α, and reduced protein levels of IL-10, an anti-inflammatory molecule (Figure 3B). Protein levels of IL-1β and NOS2 were found significantly (*p* < 0.05) higher after I/R damage in comparison with control mice, and treatment with brimonidine significantly (*p* < 0.05) counteracted the expression of these proteins (Figure 3C).

## Discussion

Glaucoma is a progressive neurodegenerative disease, and the major unmet medical need in this condition is the protection of retinal ganglion cells. In fact, it is well known that pharmacological interventions intended to only lower IOP are not always effective in preventing visual field loss, even though IOP represents the major risk factor for glaucoma progression. Neuroprotective treatment for glaucoma endeavors to preserve vision by preventing the death of RGCs. Different experimental models of ocular hypertension and different electrophysiological measurements of RGCs function have shown that cell dysfunction occurs in the early phases preceding cell death (Chou et al., 2014; Porciatti, 2015). The time lag between RGC dysfunction and death may be related both on the magnitude of IOP elevation and the susceptibility to IOP stress.

In the present study we carried out retinal I/R in mouse eye, showing that ischemic insult elicited a significant impairment of RGCs function and a remarkable expression of several inflammatory markers, such as TNF and ILs, in the retina. We also found a significant glial cells activation as demonstrated by GFAP and Iba1 upregulation.

We showed that topical treatment with brimonidine preserved RGCs function and reverted the inflammatory profile elicited by I/R injury. Further, brimonidine was able to maintain physiological levels of BDNF in the retinal tissue of I/R mice group. Relevant non-clinical studies (Yoles et al., 1999) demonstrated that brimonidine has neuroprotective properties in optic nerve degeneration and retinal ischemia (Wheeler et al., 1999) even though the authors did not figure out the mechanism of that effect. It has been hypothesized that the neuroprotection of brimonidine is related to modulation of BDNF, this latter is a potent neurotrophic factor that prevent RGCs death after axotomy in the optic nerve (Mansour-Robaey et al., 1994). Gao et al. (2002) demonstrated that brimonidine was able to up-regulate the BDNF in retinal rat after 48 h from drug treatment. How the brimonidine up-regulate retinal BDNF remains to be elucidated, in fact the authors speculated that α2 receptor activation can result in the regulation of multiple signaling pathways directly or indirectly related with BDNF expression.

It has been also demonstrated that brimonidine was able to upregulate several growth factors such as BDNF, NT3 and CTNF in ischemic rat retina (Lonngren et al., 2006). Recently, it has been demonstrated (Ortin-Martinez et al., 2014) that topical brimonidine protects retinal tissue in a light-emitting diode-induced phototoxicity. More recently, Yukita et al. (2017) showed that brimonidine enhances the electrophysiological response of RGCs through the Trk-MAPK/ERK and PI3K pathways in axotomized rat eye, hypothesizing that these pathways regulate BDNF. Beside these important proofs, another inflammatory marker, called TRAIL, has been recently highlighted. TRAIL is a member of the TNF superfamily and it is constitutively expressed in retina (Lee et al., 2002). TRAIL acts mostly through the death receptor DR5, and it is a potent mediator of prominent neuronal loss induced in both chronic and acute neurodegenerative processes, including those related to brain ischemia (Martin-Villalba et al., 1999; Cantarella et al., 2014).

Upon injury, disease or inflammation, healthy neurons may get damaged eliciting an environment alteration that activate resting microglia with a release of pro-inflammatory molecules. In addition to its pro-inflammatory pattern, microglia can also stimulate an alternative activation pathway, associated with increased production of anti-inflammatory cytokines such as IL-10 and neurotrophic factors such as BDNF to promote neuronal recovery (Di Polo et al., 1998; Gallego et al., 2012; Gonzalez et al., 2014). Privation of oxygen and nutrients during ischemia, generates reactive oxygen species production leading to inflammation. I/R injury elevates the retinal expression of several inflammatory markers such as ILs, TNF-α, TRAIL and nitric oxide (NO) (Dreyer et al., 1996; Kawasaki et al., 2000; Tezel and Wax, 2000; Wang et al., 2005). These results are in accordance with the findings generated in the present study; moreover, we observed that RGCs damage elicited the upregulation of GFAP and Iba1, demonstrating glial cells activation (Mao and Yan, 2014).

In conclusion, the ocular topical brimonidine treatment showed retinal protection in an acute model of RCGs death, reducing the expression of inflammatory cytokines, enhancing the expression of BDNF, and preserving retinal function.

## Data Availability

The raw data supporting the conclusion of this article will be made available by the authors, without undue reservation.
